# Variations in body condition score, inflammatory and metabolic biomarkers predict cognitive changes in clinically healthy senior cats

**DOI:** 10.3389/fnagi.2025.1703764

**Published:** 2025-11-05

**Authors:** Holly Memoli, Mariangela Albertini, Irit Grader, Lena Provoost, Joel Filipe, Patrizia Piotti, Paola Scarpa, Darko Stefanovski, Federica Pirrone, Carlo Siracusa

**Affiliations:** ^1^Department of Clinical Sciences and Advanced Medicine, School of Veterinary Medicine, University of Pennsylvania, Philadelphia, PA, United States; ^2^Department of Veterinary Medicine and Animal Sciences, University of Milan, Lodi, Italy; ^3^Department of Clinical Studies New Bolton Center, School of Veterinary Medicine, University of Pennsylvania, Kennett Square, PA, United States

**Keywords:** cat, behavior, cognition, aging, inflammation, inflammaging

## Abstract

**Objectives:**

The present study aimed to identify immune, metabolic, and hematological biomarkers, among those commonly monitored in clinical practice, that are predictive of age-related behavioral and cognitive changes in clinically healthy elderly cats, with the objective of highlighting potential patterns of inflammaging.

**Methods:**

A cross-sectional observational study was conducted at two veterinary institutions and involved 90 clinically healthy, privately owned domestic cats aged 7–16 years. All cats underwent physical examinations, laboratory, and behavioral screenings. Serum concentrations of the pro-inflammatory cytokine interleukin-1beta (IL-1β) and the anti-inflammatory cytokine IL-10 were measured using ELISA as markers of peripheral inflammation. Behavioral and cognitive changes were assessed using the Feline Behavioral Assessment and Research Questionnaire and Feline Cognitive Dysfunction Rating Chart, respectively. Multivariate regression analysis was used to assess the association between behavioral and cognitive outcomes and immune, metabolic, and biochemical predictors (*p* < 0.05).

**Results:**

Significant associations were identified between immune, hematological, and metabolic phenotypes indicative of chronic inflammation and cognitive changes assessed using the FCDRS Sleep–wake cycle disturbances were strongly and positively predicted by increased body condition score (BCS), alanine aminotransferase (ALT), creatinine, white blood cells (WBCs), globulin, and IL-1β levels, and negatively predicted by albumin and neutrophils. Anxiety was positively associated with higher BCS, creatinine, and IL-10, and negatively associated with IL-1β. Activity levels were positively predicted by IL-10. Altered social interactions and house-soiling were significantly associated with increased BCS.

**Conclusion and relevance:**

Findings suggest that changes in physiological parameters describing patterns of chronic inflammation are associated with measurable cognitive changes in aging cats, in the absence of overt clinical disease, which is consistent with the concept of inflammaging. Routine monitoring of standard bloodwork and BCS may offer an accessible means of tracking chronic subclinical inflammation and predicting cognitive aging in senior feline patients. These results highlight the importance of proactive cognitive screening and client education to preserve welfare and the human-animal bond in aging cats.

## Introduction

With the improvement in veterinary care, feline life expectancy has increased, subsequently resulting in an increase in the geriatric population (Ladiges, 2021). As a result, the need to prolong the health span of domestic cats has become increasingly important. While aging is a natural and gradual process, it is associated with physical, behavioral, and cognitive changes (Cordeiro et al., 2024) that generally reflects a decrease in organ and system function (Kleineidam et al., 2022) along with an enhanced risk of serious health conditions (Tenchov et al., 2024) eventually leading to various diseases and health issues that may negatively impact an individual’s quality of life. In the aging feline brain, the underlying modifications culminate in cognitive decline and a potential heightened susceptibility to develop neurodegenerative disorders such as feline cognitive dysfunction syndrome (CDS). Cognitive decline and dysfunction manifest with behavioral changes not attributable to other medical conditions (Gunn-Moore, 2011; Bellows et al., 2016a; Sordo et al., 2020), including disorientation, interaction changes, sleep-wake cycle disruption, house soiling, activity changes, and anxiety (DISHAA) (Landsberg et al., 2012). These signs frequently overlap with those of other medical conditions associated with aging, such as osteoarthritis, chronic kidney disease (CKD), hyperthyroidism, and hypertension, which complicates early recognition and diagnosis (Wrightson et al., 2023) or may be mistakenly perceived by caregivers as normal aging, potentially delaying diagnosis and intervention (Bellows et al., 2016a; Sordo et al., 2020). The behavioral changes associated with cognitive decline or dysfunction significantly impact cat welfare, diminish quality of life, and strain human-animal relationships, often leading to shelter relinquishment or euthanasia (Miller et al., 1996; Patronek et al., 1996). Given that clinical signs of cognitive decline have been reported in 28% of cats aged 11–14 and in over 50% of those aged 15 and older (Gunn-Moore, 2011; Sordo et al., 2020), the relevance of the problem is far from negligible and makes research into the mechanisms of feline brain aging and strategies to mitigate its effects urgently needed.

An increasing body of evidence from studies in laboratory, companion animals, and humans shows that age-related dysregulation in the immune response (immunosenescence), occurring even in the absence of clinical diseases, is the main responsible for these physical and behavioral changes (Costa et al., 2021; Piotti et al., 2024). Under physiological conditions, immune cells respond to pathogens or stressors by producing both pro-inflammatory and anti-inflammatory cytokines. While the former initiate immune activation, the latter restore homeostasis and prevent excessive tissue damage, ensuring a tightly regulated balance between inflammatory and anti-inflammatory responses. With age, this equilibrium becomes progressively disrupted, favoring a persistent pro-inflammatory state due to increased production of pro-inflammatory cytokines relative to their anti-inflammatory counterparts (Pirrone et al., 2024). This imbalance contributes to chronic low-grade inflammation, or “inflammaging,” which, at the brain level, increases vulnerability to neuroinflammation, possibly due to impaired synaptic plasticity, reduced neurogenesis, and the accumulation of amyloid-beta, all hallmarks of age-related cognitive decline (Sordo et al., 2020; Avondt et al., 2022; Bleve et al., 2022; Feuth, 2024).

The pro-inflammatory state associated with immunosenescence is also linked to behavioral changes such as increased anxiety, reduced activity, lethargy, social withdrawal, and altered appetite, collectively known as *sickness behaviors* (Piotti et al., 2024). Studies have highlighted that proinflammatory interleukin-1β (IL-1β) is associated with deficits in spatial memory, disrupted sleep-wake cycles, increased anxiety, and reduced social engagement in cats (Avondt et al., 2022; Piotti et al., 2024; Pirrone et al., 2024). These effects are thought to be due to IL-1β-induced neuronal damage and dysfunction, which underpin cognitive decline in aging cats (Day, 2010; Piotti et al., 2024). Understanding the behavioral manifestations of immunosenescence and linked inflammaging would enable targeted environmental and behavioral modifications to enhance the quality of life for aging cats (Pirrone et al., 2024)

Although aging is a major risk factor for numerous chronic conditions, it is important to emphasize that aging itself is not synonymous with disease, as many older individuals may maintain good physical and mental health well into advanced age. This distinction has led to growing interest in research aimed at identifying the factors that distinguish normal aging from pathological conditions, an essential step toward the early diagnosis of subtle, age-related changes (Pirrone et al., 2025). However, differentiating between physiological aging and disease can be challenging, as the threshold between the two is often unclear. The transition from an age-related physiological to a pathological process can be gradual, reflecting the progressive nature of aging itself, with early subclinical abnormalities challenging to detect. Yet it is precisely in these early, mild stages that intervention might be most effective in counteracting or at least slowing the progression toward dysfunction. Therefore, while regular veterinary assessments of aging cats, including physical examinations, behavioral evaluations and laboratory testing, are essential, the cornerstone lies in combining two complementary strategies: (1) educating caregivers to recognize and report even subtle behavioral changes in their senior cats to their veterinarian, and (2) identifying reliable biomarkers of age-related subclinical functional changes that veterinarians can easily monitor in routine clinical settings and can be predictive of cognitive decline, ideally among those biomarkers commonly measured to assess the general health status and functionality of organs subject to failure in aging cats such as liver and kidneys (Gunn-Moore, 2011; Bellows et al., 2016a).

This cross-sectional predictive study explored the relationship of a set of physiological variables commonly assessed during routine veterinary checkups, including hematological, serum biochemical parameters, and body condition score (BCS), along with serum cytokines (IL-1β and IL-10) and behavioral and cognitive changes in clinically healthy older cats (aged 7 years and above). We hypothesized that blood parameters and cytokine expression reflecting patterns of chronic inflammation would predict variations in behavioral traits and cognitive domains, even in the absence of overt clinical disease, consistent with the processes of inflammaging. Cat behavior was assessed using the Feline Behavioral Assessment and Research Questionnaire (Fe-BARQ), a validated questionnaire developed as a quantitative tool to evaluate feline behavior and behavioral problems through owner-reported surveys (Duffy et al., 2017) and increasingly adopted in feline behavioral science. Its validated framework allowed for the systematic measurement of behaviors relevant to cognitive and emotional health, including changes in activity levels, social interactions, and anxiety. The inclusion of Fe-BARQ data enabled a nuanced examination of how physiological changes, such as cytokine levels and body condition, may influence feline behavior. Moreover, its adaptability to a variety of demographic and environmental contexts ensured that the behavioral assessments were representative of real-world feline populations.

Given that there is currently no validated scale used for evaluating feline cognitive dysfunction syndrome, we chose to assess cognitive changes using the Feline Cognitive Dysfunction Rating Chart (FCDRC), the use of which has been recommended to evaluate the frequency and severity of cognitive decline-related behaviors in cats according to the DISHAA categories (Bellows et al., 2016a).

## Materials and methods

This cross-sectional observational study was conducted as part of a multicentric research project (ethical approval University of Pennsylvania IACUC protocol 807030; Regulations of the University of Milan, decision EC 29 Oct 2012, renewed under protocol No. 02-2016) designed to investigate the effects of age-related chronic inflammation on the health, behavior, and welfare of elderly cats. Data was collected using electronic questionnaires and databases (RedCap, Westlake, TX; Qualtrics, Provo, UT) to facilitate efficient survey creation, data management, and transfer to Excel (Microsoft, Redmond, WA) for statistical analysis. Identical protocols, handouts, and questionnaires were used across institutions to ensure consistency and compatibility of data, allowing seamless integration for multicentric analysis.

### Sampling

Cats were recruited for this study through multiple avenues, including opportunistic enrollment during routine wellness examinations and outreach efforts such as flyers, posters, social media platforms, and word-of-mouth communication facilitated by faculty and students at two veterinary schools. The inclusion criteria required cats to be clinically healthy, 7 years or older.

### Screening

This study employed a staged, standardized screening process to ensure the inclusion of clinically healthy cats that met standardized medical and behavioral criteria.

An extensive pre-screening protocol was implemented for all potential participants. Caregivers completed a detailed pre-screening questionnaire to gather historical and current medical and behavioral information and identify known pre-existing medical conditions or clinical signs of disease (see Supplementary Document 1). Cats meeting the inclusion criteria (Table 1) proceeded to the next stage, the medical evaluation.

**TABLE 1:** Inclusion and exclusion criteria applied during the pre-screening phase for cat selection in the study.

Inclusion criteria	Exclusion criteria
Physical	Physical
– Age ≥ 7 years – Clinically healthy determined by primary care veterinarian – Comprehensive physical examination, complete blood count, serum biochemistry, and TT4 evaluation – Absence of chronic and/or acute systemic illness or clinical signs suggestive of subclinical disease	– Uncontrolled endocrine disorders (e.g., hyperthyroidism, diabetes mellitus) – Active systemic infectious, inflammatory, or neoplastic disease – Recent surgical intervention or hospitalization within 30 days – Current use of immunosuppressive or corticosteroid therapy
Behavioral	Behavioral
– No behavioral or cognitive concerns – Not currently receiving psychopharmaceutical medications – Stable household environment for a minimum of 3 months – Demonstrates tolerance for routine veterinary handling and sample collection with minimal sedation	– Severe behavioral pathology (e.g., uncontrolled aggression, pica, self-injurious behavior) – Inability to safely undergo behavioral evaluation or diagnostic procedures – Concurrent enrollment in another psychotropic drug trial or recent initiation of behavioral pharmacotherapy (< 30 days) – Significant disruption in the home environment anticipated during the study period (e.g., relocation, new pet or household member)

### Physical and behavioral evaluation

A standardized clinical examination was performed on all cats meeting pre-screening requirements. Clinicians followed a specific checklist [adapted from Bellows et al. (2016a,b)] (see Supplementary Document 2) to ensure the cats showed no significant physical or behavioral signs of disease. Cats underwent blood sampling using minimal-stress handling techniques. Laboratory assessments included tests for Feline Leukemia Virus (FeLV), Feline Immunodeficiency Virus (FIV), heartworm, a complete blood count (CBC), serum chemistry profile, and total thyroid hormone (TT4) levels, in adherence to the minimum laboratory evaluation standards for mature, senior, and geriatric cats as outlined in the AAHA guidelines for senior pets (Epstein et al., 2005; Paepe et al., 2013). The recorded results of blood work were assessed based on the reference intervals for healthy aged cats (Bellows et al., 2016b) (see Supplementary Table 2). Fecal flotation was also performed to screen for intestinal parasites.

Behavioral evaluation was conducted using the Fe-BARQ (Feline Behavior Assessment and Research Questionnaire) to quantitatively assess feline temperament and behavior (Duffy et al., 2017). This tool evaluates behavioral traits across 23 subscales, resulting from the averaged score of one or more questions. Each question receives a score between 0 (never) and 4 (always); therefore, each subscale will also have a score within the same interval. Only subscales resulting from the score of more than one question were considered for this study, as single-question subscales were not considered robust enough to define a temperament trait. If, from the FeBARQ review, previously undisclosed behavioral concerns or signs associated with underlying pathology (e.g., excessive or generalized anxiety) emerged, the specific individual was excluded. Cats deemed healthy (i.e., free from clinically relevant signs or existing diagnoses of physical or behavioral disease, such as sensory deficits and pain), underwent cognitive assessment via questionnaire and cytokine quantification on residual serum samples.

### Cognitive evaluation

After ruling out physical and behavioral disease, cats were evaluated for cognitive decline using the Feline Cognitive Dysfunction Rating Chart (FCDRC) (Bellows et al., 2016a), a structured questionnaire developed to detect early indicators of cognitive impairment and recommended for cats aged 8 years and older (Bellows et al., 2016a). Briefly, the chart includes questions assessing the severity of behavioral changes, grouped into five domains: disorientation and memory, interactions, sleep–wake cycle, house-soiling, activity, and anxiety. Each behavior is rated on a four-point scale (0 = none to 3 = severe), with higher scores indicating greater impairment. Each FCDRC domain is evaluated independently, and no FCDRC total score is proposed or validated in Bellows et al. (2016a). We followed the same approach in using the FCDRC in this study.

Although the FCDRC was developed to screen for cognitive dysfunction, emerging evidence suggests that early changes in these domains may precede and predict pathological cognitive decline. Studies in dogs (Bain et al., 2001) and humans (Albert, 2011) have shown that subtle cognitive changes can overlap with and anticipate later dysfunction. Therefore, we employed this clinical screening tool to assess early cognitive changes in our population of healthy senior cats.

### Cytokine quantification

Serum levels of IL-1β and IL-10 were quantified using commercially available ELISA kits specifically designed for quantitative determination in cats (Invitrogen, Thermo Fisher Scientific, Frederick, MD). Serum samples were collected and immediately stored at −20 °C in accordance with the manufacturer’s instructions, where they remained for up to 6 months without thawing. Prior to analysis, samples were thawed at room temperature and processed by a laboratory technician who was blinded to the study. ELISAs were performed following the manufacturer’s protocols. All standards and samples were diluted 1:2 or 1:4 based on preliminary testing and plated in duplicate on 96-well plates coated with feline IL-1β or IL-10 antibodies. After overnight antigen binding at 4 °C, plates were washed, and a biotin-conjugated antibody specific to IL-1β or IL-10 was added, followed by a one-hour incubation at room temperature. Plates were then washed, and streptavidin-HRP was added, with a subsequent 45-min incubation at room temperature. After another wash, the chromogen substrate (TMB) was added, and the plates were incubated in the dark at room temperature for 30 min. The reaction was stopped with a stop solution, and absorbance was read at 450 nm using a spectrophotometer (Molecular Devices, SpectraMax ID3, San Jose, CA). Each sample was run in duplicate, and all samples were analyzed twice to ensure accuracy. Cytokine concentrations were calculated based on standard curves generated for IL-1β (16–4,000 pg/mL) and IL-10 (0.2–50 ng/mL). The average intra-assay coefficient of variation was 3.83 % for IL-1β and 3.06 % for IL-10, while the assay sensitivity was 26.02 pg/mL and 0.08 ng/mL, respectively.

### Statistical analysis

All analyses were conducted using Stata 18, StataCorp, College Station, TX, with two-sided tests of hypotheses and a *P*-value < 0.05 as the criterion for statistical significance. Descriptive analyses included computation of medians and interquartile ranges of continuous variables and tabulation of categorical variables. Tests of normal distribution (Shapiro-Wilk test) were performed to determine the extent of skewness of the data. Frequency counts and percentages were used to summarize categorical variables (e.g., sex and location of data collection).

In accordance with the original scoring method, FeBARQ scores for sections that were missing more than 25% of entries were not calculated (Duffy et al., 2017).

Inference statistical analysis was conducted in three steps. First, Spearman rank correlation analysis was used to select independent variables potentially acting as confounding factors and predictors associated with the outcome variables FeBARQ scores and FCDRC scores. The threshold was set at a *P*-value < 0.2, indicating a trend of pairwise association (Hosmer et al., 2013; Tukey, 1977). Second, the lasso Poisson regression estimation procedure was used to identify a subset of independent variables that showed the strongest association with the outcome of interest (Hastie et al., 2015). Third and final, mixed effects Poisson regression was used to assess the association between fixed effects of the treatment and the predictors and confounders identified in the previous step, with random effects set at the level of individual animals.

## Results

**FIGURE 1:**
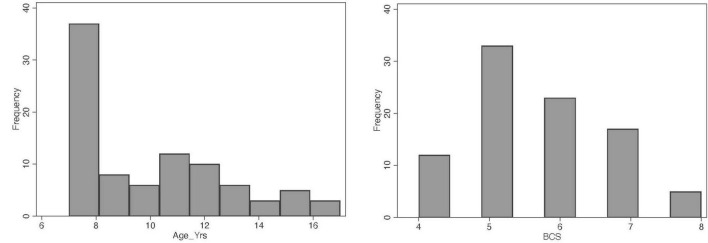
Distribution of the variables age and body condition score (BCS) in the population of cats studied.

A total of 169 cat owners were initially contacted for potential enrollment in the study, of which 156 agreed to participate and filled out the pre-screening questionnaire. Following pre-screening, 97 cats met the initial eligibility criteria. However, seven cats were excluded based on clinical examination and laboratory findings, resulting in a final sample of 90 cats included in the analyses. Of these, cytokine measurements were completed for 75 cats, the FCDRC was completed by caregivers for 85 cats, and the Fe-BARQ behavioral questionnaire was completed for 82 cats (see Supplementary Tables 1, 2 for further details). In the final sample (*n* = 90), 51 were females and 39 males, aged 7–16 years old (Figure 1), with a median of 9.3 years. All cats were neutered. Most of the cats (*n* = 79) were Domestic Shorthair. Other represented breeds included Maine Coon (*n* = 3), British Shorthair (*n* = 2), Persian (*n* = 2), and one each of Domestic Longhair, Ragdoll, and Siamese. The body condition score (BCS) of the cats ranged from 4 to 8 (Figure 1), with a median value of 5.5. Descriptive statistics for FCDRC severity scores are reported in Table 2.

**TABLE 2:** Descriptive statistics of the Feline Cognitive Dysfunction Rating Chart (FCDRC) severity scores (*n* = 85 cats).

Parameter	Mean ± SE
	IQR (25th–75th percentile)
Disorientation and memory	1.28 ± 0.08
	0.21 (1.00–1.21)
Interactions	2.12 ± 0.23
	2 (1.00–3.00)
Sleep-wake cycles	2.25 ± 0.25
	1.50 (1.00–2.50)
House-soiling	1.81 ± 0.24
	0.00 (1.00–1.00)
Activity	1.73 ± 0.18
	0.60 (1.00–1.60)
Anxiety	2.44 ± 0.33
	2.00 (1.00–3.00)

The score for each parameter ranges between 0 and 3. Values are presented as mean ± standard error (SE) and interquartile range (IQR; 25th–75th percentile).

Several independent variables showed a trend of association (*P* < 0.2) in the Spearman rank analysis. Based on these correlations, we included the variables sex, age, and location of data collection in the Lasso Poisson regression models as confounding factors. The following variables were included in the regression models as predictors: body condition score (BCS), IL-1β, blood urea nitrogen (BUN), creatinine, alanine aminotransferase (ALT), aspartate transferase (AST), alkaline phosphatase (ALP), albumin, globulin, total white blood cells (WBC), neutrophils, lymphocytes, and total T4.

The Lasso Poisson regression estimation procedure delivered strong associations (*P* < 0.05) of independent variables with the outcome variables FeBARQ-Section 10 (Familiar Cat Aggression), FeBARQ-Section 19 (Excessive/Compulsive Self Grooming), FCDRC-Disorientation and Memory, FCDRC-Interactions, FCDRC-Sleep-Wake Cycle, FCDRC-House-soiling, FCDRC-Activity, FCDRC-Anxiety, and FCDRC-Overall Score. We therefore included the models for these outcome variables in the third step of our analysis, the mixed effects Poisson regression.

**FIGURE 2:**
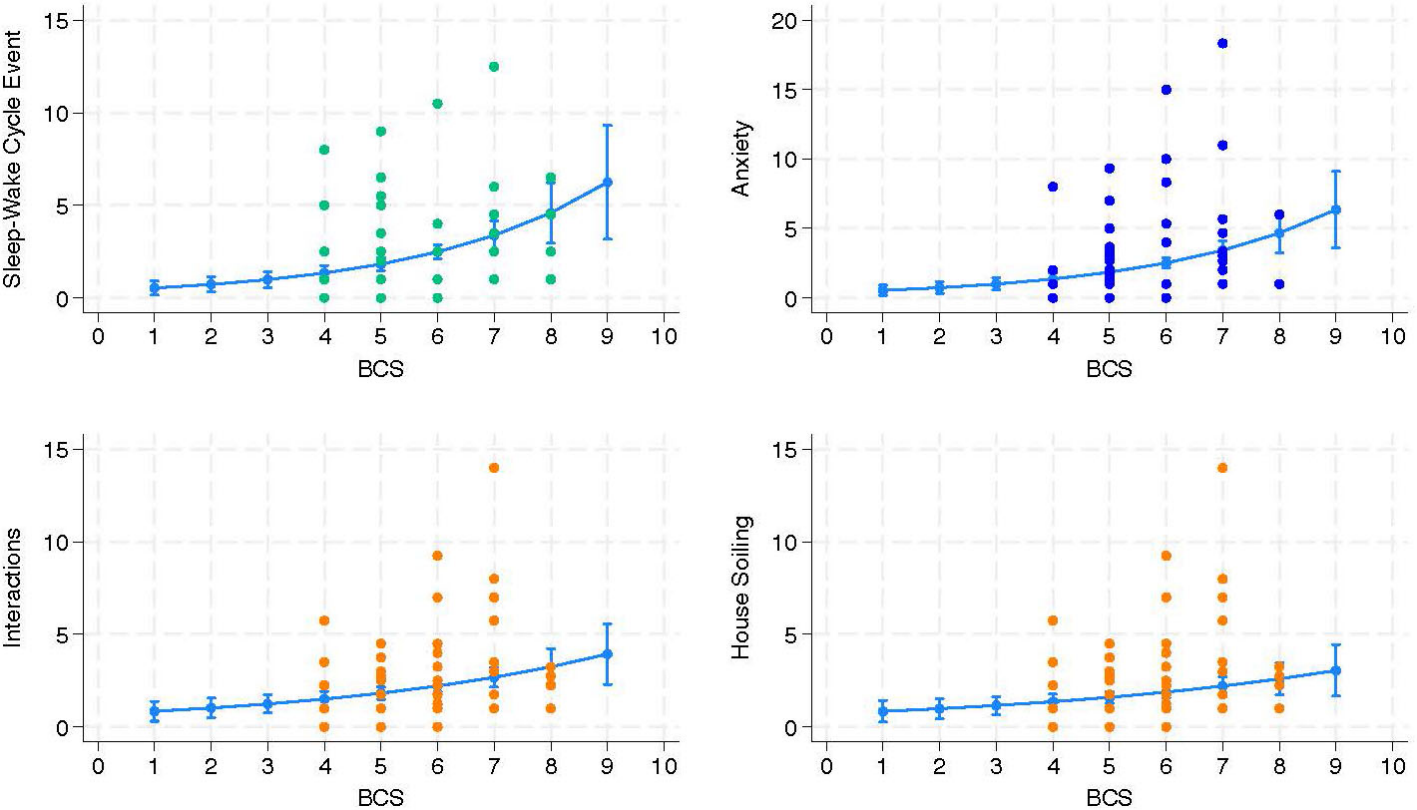
The independent variable body condition score (BCS) was significantly associated with and positively predicted changes in the three Feline Cognitive Dysfunction Rating Chart (FCDRC) outcome variables Sleep-Wake Cycle, Anxiety, Interactions, and House-soiling (*n* = 90 cats).

Upon review of the models delivered by the mixed effects Poisson regression, no predictors were identified for the two FeBARQ subscales included. Conversely, a review of the FCDRC scores revealed that Sleep-Wake Cycle, Anxiety, Interactions, Activity, and House-soiling were significantly associated with specific blood markers and BCS (see Table 3). Specifically, higher Sleep-Wake Cycle scores, which indicate greater disruption, were positively predicted by BCS, globulins, ALT, ALP, creatinine, WBC, and IL-1β, and negatively predicted by neutrophils and albumins. After adjusting for the confounding variables, sex, and location of the data collection, anxiety-related behaviors were positively predicted by higher BCS, creatinine, and IL-10, and negatively predicted by IL-1β. BCS was a positive predictor of higher scores in interactions, which indicates greater alteration, while IL-10 positively predicted more severe alterations in activity after adjusting for age. BCS was also a positive predictor of house-soiling after having adjusted for the effect of the location of data collection. No significant associations were found with the Disorientation and Memory dimension of the FCDRC. It is of particular interest that BCS was directly associated with four of the five FCDRC independent variables (Figure 2). A one-unit rise in BCS resulted in a 36% higher likelihood for an increase in the frequency of Sleep-Wake Cycle, 35% higher likelihood in Anxiety, 21% higher likelihood of an increase in the frequency of Interactions, and 22% higher likelihood of increased house-soiling (see Table 3).

**TABLE 3:** Significant predictors from the multivariate analysis for cognitive domains assessed by the Feline Cognitive Dysfunction Rating Chart (FCDRC).

Independent variable	IRR	z	*P*(z)	95% CI min	95% CI max	Min	Max	Median
**Sleep-wake cycle (*n* observations = 72)**								
BCS	1.36	4.13	0.001	1.17	1.57	4	8	5.5
Albumin (g/dl)	0.46	−2.53	0.011	0.26	0.84	2.7	4.7	3.7
Globulin (g/dl)	1.44	2.50	0.012	1.08	1.93	2.4	5.5	3.8
ALT (U/l)	1.01	3.27	0.001	1.00	1.01	27	178	52
ALP (U/l)	1.00	2.02	0.044	1.00	1.01	8	131	39
Creatinine (mg/dl)	2.80	3.98	0.001	1.68	4.65	0.8	3.2	1.6
WBC (× 10∧3/μl)	1.28	3.30	0.001	1.10	1.49	3.23	18.1	6.93
Neutrophils (× 10∧3/μl)	0.72	−3.41	0.001	0.60	0.87	1.63	13.9	4.3
IL1 β (pg/ml)	1.00	3.02	0.003	1.00	1.00	16	4,000	423.3
**Anxiety (*n* observations = 75)**								
BCS	1.35	4.25	0.001	1.17	1.55	4	8	5.5
Creatinine (mg/dl)	1.87	3.15	0.002	1.26	2.77	0.8	3.2	1.6
IL10 (ng/ml)	1.02	2.23	0.006	1.00	1.04	0.2	50	0.658
IL1 β (pg/ml)	0.99	−2.51	0.012	0.99	0.99	16	4,000	423.3
Sex - male	0.73	−2.06	0.040	0.53	0.98	–	–	–
Location - Milan	0.55	−2.97	0.003	0.37	0.82	–	–	–
**Activity (*n* observations 75)**								
IL10 (ng/ml)	1.01	2.19	0.028	1.00	1.03	0.2	50	0.658
Age	1.08	2.45	0.014	1.02	1.15	7	18	10
**Interactions (*n* observations = 90)**								
BCS	1.21	2.99	0.003	1.06	1.37	4	8	5.5
**House-soiling (*n* observations = 90)**								
BCS	1.22	2.85	0.004	1.06	1.40	4	8	5.5
Location - Milan	2.02	4.31	0.001	1.47	2.78	–	–	–

IRR, incidence rate ratio; z, z-statistic; P(z), *p*-value associated with the z-statistic; 95% CI, 95% confidence interval (min−max); Min, Max, and Median represent the minimum, maximum, and median observed values for each independent variable; BCS, body condition score. (*n* = 90 cats).

## Discussion

In this study, we identified several biological variables, including serum concentrations of both pro- and anti-inflammatory cytokines (IL-1β and IL-10), biochemical parameters (albumin, globulins, ALT, ALP, and creatinine), hematological variables (WBCs, neutrophils) and BCS, that predicted, either independently or in combination, five cognitive domains (sleep–wake cycle, social interactions, anxiety, activity, and house-soiling) assessed using the feline cognitive disfunction rating chart (FCDRC) in a population of cats aged 7 years and older. The trends of these associations are consistent with patterns typically observed in chronic inflammatory conditions. Importantly, all cats were deemed clinically healthy based on physical examination and routine laboratory screening, with all values falling within known age-appropriate reference intervals, except for cytokines, for which no clinically validated cutoffs or established baseline reference values currently exist in cats. Therefore, these findings suggest that biological signals may co-vary with—or potentially influence—cognitive function, outlining multidimensional profiles indicative of increased risk for inflammation-related cognitive changes in aging cats, in the absence of overt clinical signs of inflammatory disease detectable at a routine medical examination.

Variations in biomarkers potentially indicative of a pattern of low-grade inflammation (i.e., increases in globulins, WBCs, and IL-1β, and decreases in albumin and neutrophils) were associated with a higher likelihood of sleep–wake cycle disturbances. Elevated IL-1β has been linked to sleep deprivation in humans (Feuth, 2024), which reinforces the relationship between inflammation and sleep alterations across species. One possible explanation for this association lies in the dynamic interplay between the immune system and sleep architecture. Physiological sleep involves a shift from T helper 1-driven pro-inflammatory activity, marked by cytokines like IL-1β that promote NREM sleep, to a T helper 2-dominant, regulatory phase characterized by anti-inflammatory cytokines such as IL-10, which support immune resolution and sleep recovery (Feuth, 2024). In aging individuals, including humans and cats (Sordo et al., 2020), this balance may be disrupted: sleep disturbances can elevate systemic inflammation, while ongoing inflammation, even at a low grade, may impair sleep regulation. The observed association between IL-1β levels and sleep–wake cycle disturbances in clinically healthy senior cats may thus reflect underlying mechanisms of inflammaging, driven by this bidirectional relationship between inflammation and sleep alterations.

Albumin, a negative acute-phase protein, is expected to decrease in the presence of chronic inflammation and is often inversely correlated with elevated levels of pro-inflammatory cytokines like IL-1β (Dinarello, 1984). Consistently, in our study, lower serum albumin concentrations significantly predicted greater disturbances in the sleep–wake cycle domain, supporting a role for chronic inflammatory processes in sleep regulation impairments in aging cats. Conversely, the behavior of neutrophils and other white blood cells in aging individuals, as part of immunosenescence, is not clear and consistent. Aging in mice, dogs, and humans has been associated with neutrophilia, neutropenia, or a stable number of circulating neutrophils. Several factors, such as a shift in the hematopoietic stem cells, margination of blood cells, expression of other cytokines, and changes in the gut microbiota, can explain the complexity of this response (Day, 2010; Avondt et al., 2022; Gao et al., 2024), which undoubtedly warrants further clarification.

As expected, increases in ALT, ALP, and creatinine, as well as higher BCS, also significantly predicted sleep–wake cycle disruptions in our study. Elevated hepatic enzymes and creatinine are often typical markers of chronic physical conditions, often subclinical, commonly seen in aging cats, such as organ dysfunction (kidney and liver insufficiency), and systemic low-grade inflammation (Denenberg et al., 2024). Age-related increases in liver enzyme levels are also commonly reported in clinically healthy dogs (Kusaba et al., 2024). Similarly, a positive association of BCS with low-grade chronic inflammation and a higher prevalence of chronic inflammatory diseases has been documented in both dogs (Frank et al., 2015) and cats (Teng et al., 2018). This association is largely attributed to the metabolically active role of white adipose tissue that promotes the production of pro-inflammatory mediators and induces lipotoxicity through adipokines, increasing the risk of overweight and obese individuals for inflammation-related comorbidities, some potentially life-threatening (Vitor et al., 2024; Rowe et al., 2015; Wallis and Raffan, 2020; Clark and Hoenig, 2021). In clinical practice, the prevalence of overweight and obesity has significantly increased in the feline population (Saavedra et al., 2024) and a positive association with age has been clearly detected (Teng et al., 2017; Wall et al., 2019; Arena et al., 2021), posing a growing concern. Although previous research has explored the connection between BCS and behavior in cats (De Godoy and Shoveller, 2017), it primarily focused on physical activity and caregiver interactions in the context of feeding. This is the first study to directly link BCS with cognitive changes. In our study, cats had a median BCS of 5.5 (range: 4–8) on a 9-point scale, which is often considered consistent with a healthy weight range for the age group involved (World Small Animal Veterinary Association, 2020; Teng et al., 2018; Saavedra et al., 2024).

The cats with lower IL-1β had increased odds of exhibiting more severe anxiety, as assessed by the FCDRC, whereas IL-10 emerged as a positive predictor of greater severity of changes in both the anxiety and activity domains. It may be worth noting that, in the FCDRC, anxiety was assessed through questions about anxious, fearful, or phobic responses to specific stimuli, including people, noises, or places (Bellows et al., 2016a). Activity, on the other hand, was evaluated through questions addressing changes in behaviors, some of which, such as playfulness, grooming habits, exploration, and overall interest in food, may be influenced by anxiety or emotional distress. The link between inflammation and fear and anxiety has been documented in several species, including laboratory rodents, dogs, and humans (Peirce and Alviña, 2019; Gilbert et al., 2025), functioning as highly conserved defense mechanisms, which help an organism adapt and survive, under physiological conditions (Nelles et al., 2025;, Ashley et al., 2012; Widiger and Oltmanns, 2017). In a well-regulated inflammatory response, cytokines can modulate amygdala circuits involved in anxiety regulation (Lee et al., 2025), while anxiety itself enhances survival by increasing vigilance and promoting responses to potential threats. Co-variations between cytokine concentrations and fear- or anxiety-related behaviors support an organism’s capacity to adapt to internal and/or external stressors through the activation of homeostatic and allostatic mechanisms (Widiger and Oltmanns, 2017), essentially reflecting a state of immune-behavioral readiness. In our study, the positive association of anti-inflammatory, neuroprotective, and emotional regulatory IL-10 (Patel et al., 2021; Ying et al., 2023) with anxiety and activity, along with the inverse association between IL-1β and anxiety, may represent this regulatory balance and buffered inflammatory load in these aging cats.

This delicate balance between activation and regulation may, however, become increasingly disrupted with age (Shive and Pandiyan, 2022) with the anti-inflammatory response undergoing dysregulation and losing its compensatory capacity (Wautier and Wautier, 2023). One potential driver of this shift is the cumulative effect of repeated real or perceived stressors and recurrent low-level bouts of inflammation experienced throughout life (Chiang et al., 2022). Over time, this allostatic load and its more severe form, allostatic overload (Guerra et al., 2021) can promote the persistent, low-grade inflammation characteristic of aging (inflammaging). This, in turn, accelerates the aging process (De La Fuente, 2008) by contributing to chronic anxiety and stress, and various diseases, including metabolic disorders (Alotiby, 2024). This may help explain the positive association observed in our study between anxiety levels and two metabolic markers, BCS and creatinine, both of which, when elevated, may signal underlying chronic inflammatory processes involving altered energy homeostasis or early renal functional changes, respectively. Interestingly, serum creatinine concentration is the classical biomarker of chronic kidney disease (CKD) in cats (Kongtasai et al., 2022; Mortier et al., 2023), a condition known to be associated with both systemic inflammation (Uva et al., 2023) and chronic stress-related cognitive changes, including altered sleep duration (Sordo et al., 2020) and increased anxiety (Kim et al., 2025), particularly in frail populations such as the elderly (Dziubek et al., 2016). Early diagnosis remains particularly challenging, due to both the limitations of conventional biomarkers such as creatinine itself (Kongtasai et al., 2022) or the absence of evident clinical signs until CKD has advanced (Boyd et al., 2008). As a result, there is a well-recognized need for novel indicators capable of detecting renal dysfunction or predicting its progression before conventional clinical and behavioral signs become apparent (Kongtasai et al., 2022). Given that cognitive changes can precede the onset of systemic signs of illness in aging animals (Pirrone et al., 2024), our finding of a positive association between increasing creatinine levels and anxiety supports the idea that cognition-related behavioral changes, particularly those linked to emotional states such as anxiety or fear, might serve as useful early cues of subclinical renal insufficiency, a condition that is likely to progress into chronic dysfunction, which remains one of the leading causes of morbidity and mortality in cats (Uva et al., 2023).

Body condition score also emerged as a robust positive predictor of altered social interactions, which in the FCDRC include behaviors such as social withdrawal, clinginess combined with irritability, inappropriate vocalizations, and altered relationships with other pets in the household. It is plausible that this relationship, too, may be at least partially mediated by an underlying state of low-grade chronic inflammation, with immune system activity affecting the central nervous system, thereby influencing emotional regulation and stress responsiveness (Gorzelanna and Miszczak, 2024). Indeed, a previous study using the Fe-BARQ questionnaire also identified an association between chronic inflammatory disease and anxiety-related behaviors in cats. These included excessive grooming and increased fear of novelty, as well as heightened etepimeletic (care-soliciting) behaviors, such as purring and greater sociability toward humans, compared to healthy cats, in a population with a mean age of 8 years (Gilbert et al., 2025). The association between increased BCS and anxiety, together with the possible underlying low-grade inflammatory state mentioned above, can help make sense of the positive predictive value that BCS has for house-soiling. House-soiling may be a sign of anxiety (Barcelos et al., 2018), and both phenomena have been independently linked to significant social stressors in cats, such as those arising in multi-cat households (Finka and Foreman-Worsley, 2022). Moreover, increased body condition has been associated with age-related conditions involving chronic pain, particularly lameness (Scarlett and Donoghue, 1998), degenerative joint disease, and osteoarthritis (Hardie et al., 2002), which are among the potential contributors to house-soiling (Learn and Horwitz, 2024). Although the senior cats in our study were considered clinically healthy and did not show overt signs of lameness and pain at home and during the examination, no specific diagnostic imaging (e.g., radiographs) was performed to rule out these conditions. Therefore, the presence of such disorders at a subclinical level cannot be entirely excluded. In addition, we opted not to use a complete structured owner-based assessment for chronic orthopedic pain, such as the checklist provided by Enomoto et al. (2020), to minimize the time spent by the participants filling in questionnaires and, therefore, maximize recruitment and retention. However, we took care to include most of the behaviors on this list, such as the ability to jump, climbing stairs, and playfulness, in our prescreening (see Supplementary Document 1) and screening process.

Variations of the BCS in cats 7 years and older have been associated with age and sex of the animals, with the BCS showing a modest increase between 7 and 10 years, particularly in male cats, and then decreasing (Pye et al., 2025). The composition of the population studied, with a median age below 10 years and a majority of female cats, may have influenced our findings. However, none of our significant statistical models returned an interaction of BCS with age, and only the model FCDRC Anxiety included an inverse interaction between BCS and Sex-Male (Table 3).

It is worth noting that none of the factors in our study were found to be predictive of behavior changes measured using the FeBARQ questionnaire. In previous studies, this tool has proved sensitive enough to detect owner-reported behavior differences among breeds and changes associated with chronic inflammatory disease (Wilhelmy et al., 2016; Gilbert et al., 2025). On the other hand, several behavior changes measured via the FCDRC were associated with factors included in this study, as discussed above. This difference may be explained by the use of selected behavior changes (DISHAA) that are clinically related to cognitive changes and underscore the need for veterinarians to proactively question caregivers about behaviors that are typically associated with cognitive decline, rather than inquire about generic behavior changes observed. Although the FCDRC is based on research and clinical findings and its use has been recommended to screen for cognitive changes in senior cats (Bellows et al., 2016a,b), no questionnaire or chart, including the FCDRC, has been validated to assess cognitive changes in cats. Future research aimed at validating this chart or developing standardized assessment tools will be essential to strengthen the robustness of studies on feline cognitive aging.

The results of this study may have been affected by the inherent complexity and variability of the inflammatory response. Cytokines are increasingly studied as biomarkers of inflammaging and chronic disease risk (Koelman et al., 2019), but their measurement is challenging due to fluctuations caused by stress, feeding, and circadian rhythms (Kim and Maes, 2003; Yamakawa et al., 2015). Moreover, undetected subclinical conditions in the sampled cats may have contributed to variability not accounted for in a one-time clinical assessment. Nonetheless, previous research indicates that single measurements of selected cytokines, such as IL-10, can reflect stable individual levels over time (Koelman et al., 2019), supporting their potential use in longitudinal studies. Given that early physical, behavioral, and cognitive changes have been observed in apparently healthy aging cats (Epstein et al., 2005; Paepe et al., 2013; Bellows et al., 2016a; Dhaliwal et al., 2023; Pirrone et al., 2024) future large-scale, prospective studies, including serum cytokine measurements, particularly those which, based on current evidence, can serve as reliable indicators of an individual’s long-term inflammatory status (Koelman et al., 2019) are needed to capture even subtle pathophysiological changes in inflammatory response and identify individuals at increased risk.

## Conclusion

This study provides preliminary evidence identifying cytokine, hematologic, and metabolic markers associated with cognitive alterations, reflecting patterns of inflammaging in healthy elderly cats. One of the primary objectives of this study was to provide veterinarians and caregivers with tools to recognize inflammaging-related dynamics in senior cats at an early stage by detecting biological, behavioral, and cognitive changes before they progress to overt dysfunction. Regardless of the direction of causality, which cannot be determined due to the cross-sectional design and the correlational nature of the analyses, these findings support a biobehavioral model in which physiological and emotional pathways contribute to brain aging in cats. As these patterns may become dysregulated with age, both leading to and resulting from chronic inflammation-related conditions, which often present with subtle early cognitive changes, identifying a combination of markers across these domains that could serve as early clinical indicators of cognitive decline may represent a cornerstone for effective targeted preventive strategies to support brain aging and improve the overall welfare of geriatric feline populations.

While cytokines are not commonly measured in clinical practice to monitor inflammatory processes, our findings suggest that other biomarkers, namely serum ALT, ALP, creatinine, albumin, globulins, and BCS, which are routinely assessed during standard veterinary visits, may serve as useful indicators of inflammaging when interpreted along with cognitive function in cats. If serum biomarkers cannot be monitored by cat caregivers at home and should therefore be frequently measured by the veterinary clinician during bi-annual checkups, BCS can be easily and at no cost monitored (Vitor et al., 2024) at home by cat caregivers. Veterinarians should educate cat caregivers on the importance of regularly assessing and maintaining an appropriate BCS in their cat, especially in aging animals, to help prevent or delay the onset of cognitive decline. In addition, caregivers should be trained to observe and recognize any cognitive changes, which should then be actively investigated by the veterinarians through simple screening tools like the cognitive questionnaire adopted in this and other studies. This is particularly relevant for two reasons: first, because cognitive changes, especially when combined with clinical biomarkers, may help identify, at an early stage, profiles consistent with underlying inflammaging; and second, because some of these changes, such as disruptions in the sleep–wake cycle or increased anxiety, can significantly impact both animal welfare and caregiver wellbeing, straining the human–animal bond and increase the risk of rejection or abandonment of the aging cat. Early identification is therefore essential to enable more timely and effective interventions.

## Data Availability

The raw data supporting the conclusions of this article will be made available by the authors, without undue reservation.
