# Supplementary material for: Variations in body condition score, inflammatory and metabolic biomarkers predict cognitive changes in clinically healthy senior cats
Source: Front Aging Neurosci. 2025 Nov 5;17:1703764. doi: 10.3389/fnagi.2025.1703764 (PMC12627069; doi:10.3389/fnagi.2025.1703764)
Supplement: Supplementary file 1 [file Table_1.doc]

**Supplementary Table 1**. Signalment and procedures completed for each of the 90 cats (e.g., C-1 to C-90) enrolled in the study. The cats were mostly Domestic Shorthair (n = 79); therefore, breed was excluded from the table. Physical examinations were completed for all 90 cats. A Complete Blood Cell (CBC) count and a serum chemistry panel were obtained for 82 cats, and cytokine measurements for 75 cats. The owners of 82 cats completed the Feline Behavior Assessment and Research (FeBARQ) questionnaire, and 85 owners completed the Feline Cognitive Dysfunction Rating Chart (FCDRC).

| **Number of Cats** | **Age (years)** | **Sex** | **Physical Examination** | **Bloodwork** | **Cytokines** | **FCDRC** | **FeBARQ** |
| --- | --- | --- | --- | --- | --- | --- | --- |
| C-1 | 11 | Female | Y | Y | Y | Y | Y |
| C-2 | 7 | Male | Y | Y | Y | Y | Y |
| C-3 | 15 | Male | Y | Y | Y | Y | Y |
| C-4 | 10 | Female | Y | Y | Y | Y | Y |
| C-5 | 7 | Female | Y | N | N | Y | Y |
| C-6 | 9 | Female | Y | Y | Y | Y | Y |
| C-7 | 13 | Female | Y | Y | Y | Y | Y |
| C-8 | 13 | Female | Y | Y | Y | Y | Y |
| C-9 | 15 | Female | Y | Y | Y | N | Y |
| C-10 | 8 | Female | Y | Y | Y | Y | Y |
| C-11 | 12 | Male | Y | Y | Y | Y | Y |
| C-12 | 13 | Female | Y | N | N | Y | N |
| C-13 | 7 | Female | Y | Y | Y | Y | Y |
| C-14 | 7 | Male | Y | Y | Y | Y | Y |
| C-15 | 9 | Female | Y | Y | Y | Y | Y |
| C-16 | 7 | Female | Y | Y | Y | Y | Y |
| C-17 | 9 | Male | Y | Y | Y | Y | Y |
| C-18 | 11 | Male | Y | Y | Y | Y | Y |
| C-19 | 8 | Male | Y | Y | Y | Y | Y |
| C-20 | 7 | Female | Y | Y | Y | Y | Y |
| C-21 | 11 | Male | Y | Y | Y | Y | Y |
| C-22 | 12 | Female | Y | Y | Y | Y | Y |
| C-23 | 12 | Female | Y | Y | Y | Y | Y |
| C-24 | 8 | Male | Y | Y | Y | Y | Y |
| C-25 | 10 | Female | Y | Y | Y | N | Y |
| C-26 | 7 | Female | Y | Y | Y | Y | Y |
| C-27 | 10 | Male | Y | N | N | Y | N |
| C-28 | 8 | Female | Y | Y | Y | Y | Y |
| C-29 | 11 | Male | Y | Y | N | Y | Y |
| C-30 | 8 | Male | Y | Y | Y | Y | Y |
| C-31 | 13 | Female | Y | Y | Y | Y | Y |
| C-32 | 12 | Male | Y | Y | Y | Y | Y |
| C-33 | 8 | Female | Y | Y | Y | Y | Y |
| C-34 | 7 | Female | Y | Y | Y | Y | Y |
| C-35 | 11 | Male | Y | Y | Y | Y | Y |
| C-36 | 8 | Male | Y | Y | Y | Y | Y |
| C-37 | 8 | Female | Y | Y | Y | Y | Y |
| C-38 | 7 | Male | Y | Y | Y | Y | Y |
| C-39 | 8 | Female | Y | Y | Y | Y | Y |
| C-40 | 7 | Female | Y | Y | Y | Y | Y |
| C-41 | 8 | Male | Y | Y | Y | Y | Y |
| C-42 | 7 | Male | Y | Y | Y | Y | Y |
| C-43 | 12 | Female | Y | Y | Y | Y | Y |
| C-44 | 9 | Male | Y | Y | Y | Y | Y |
| C-45 | 7 | Female | Y | Y | Y | Y | Y |
| C-46 | 10 | Female | Y | Y | Y | Y | Y |
| C-47 | 11 | Female | Y | Y | Y | Y | Y |
| C-48 | 7 | Male | Y | Y | Y | Y | Y |
| C-49 | 12.5 | Female | Y | Y | Y | Y | Y |
| C-50 | 14 | Female | Y | Y | Y | Y | Y |
| C-51 | 14 | Male | Y | Y | Y | Y | Y |
| C-52 | 7 | Male | Y | N | N | Y | N |
| C-53 | 7 | Female | Y | N | N | Y | N |
| C-54 | 8 | Male | Y | Y | Y | Y | Y |
| C-55 | 8 | Male | Y | Y | Y | Y | Y |
| C-56 | 12 | Female | Y | Y | Y | Y | Y |
| C-57 | 8 | Female | Y | Y | Y | Y | Y |
| C-58 | 8 | Male | Y | Y | Y | Y | Y |
| C-59 | 9.5 | Male | Y | Y | Y | Y | Y |
| C-60 | 11 | Male | Y | N | N | Y | N |
| C-61 | 13 | Male | Y | Y | N | Y | Y |
| C-62 | 11 | Male | Y | Y | Y | Y | Y |
| C-63 | 7 | Male | Y | Y | Y | Y | Y |
| C-64 | 9 | Female | Y | Y | Y | Y | Y |
| C-65 | 12.5 | Female | Y | Y | Y | Y | Y |
| C-66 | 11 | Female | Y | Y | Y | Y | Y |
| C-67 | 7 | Male | Y | N | N | Y | Y |
| C-68 | 11 | Female | Y | Y | Y | Y | Y |
| C-69 | 8 | Female | Y | N | N | Y | Y |
| C-70 | 9 | Female | Y | Y | Y | Y | Y |
| C-71 | 8.5 | Male | Y | Y | Y | Y | Y |
| C-72 | 17 | Male | Y | Y | Y | Y | Y |
| C-73 | 12 | Female | Y | Y | N | Y | Y |
| C-74 | 8 | Female | Y | Y | N | Y | Y |
| C-75 | 13 | Male | Y | Y | Y | Y | Y |
| C-76 | 16 | Female | Y | Y | Y | Y | Y |
| C-77 | 12.5 | Male | Y | Y | Y | N | Y |
| C-78 | 7 | Female | Y | Y | Y | N | Y |
| C-79 | 15 | Female | Y | Y | N | Y | Y |
| C-80 | 15 | Female | Y | Y | Y | Y | Y |
| C-81 | 16 | Female | Y | Y | Y | Y | Y |
| C-82 | 8 | Female | Y | Y | N | Y | Y |
| C-83 | 8.5 | Female | Y | Y | N | Y | Y |
| C-84 | 10 | Female | Y | Y | Y | Y | Y |
| C-85 | 7 | Male | Y | Y | Y | Y | Y |
| C-86 | 11 | Female | Y | Y | Y | Y | Y |
| C-87 | 15 | Male | Y | Y | Y | Y | Y |
| C-88 | 7 | Female | Y | Y | Y | Y | N |
| C-89 | 14.5 | Male | Y | Y | Y | N | N |
| C-90 | 11 | Male | Y | Y | Y | Y | N |
