# Supplementary material for: Variations in body condition score, inflammatory and metabolic biomarkers predict cognitive changes in clinically healthy senior cats
Source: Front Aging Neurosci. 2025 Nov 5;17:1703764. doi: 10.3389/fnagi.2025.1703764 (PMC12627069; doi:10.3389/fnagi.2025.1703764)
Supplement: Supplementary file 2 [file Table_2.doc]

**Supplementary Table 2**: Values and reference intervals for the hematological, biochemical, and inflammation parameters in the sample of cats in the study (n = 82 cats for all values, except for cytokines with n = 75). Values are presented as mean ± standard error (SE) and interquartile range (IQR; 25th–75th percentile). SE indicates the variability of the sample mean, whereas the IQR represents the range containing the central 50 % of values.

| Parameter | Mean ± SE  IQR (25th–75th percentile) | Lab reference Intervals | Reference intervals for healthy aged cats according to Bellows et al^1^  7–10 years ≥11 years | |
| --- | --- | --- | --- | --- |
| WBC (x 10^3/μl) | 7.25 ± 0.32  3.00 (5.46 - 8.46) | 5.10-16.20 | 3.10-16.20 | 3.70-16.40 |
| Neutrophils (x 10^3/μl) | 4.39 ± 0.24  1.92 (3.24 - 5.16) | 2.30-11.60 | 1.5-11.0 | 2.00-11.40 |
| Lymphocytes (x 10^3/μl) | 2.19 ± 0.16  1.52 (1.27 – 2.79) | 0.90-6.00 | 0.60-5.10 | 0.50-4.80 |
| Albumin (g/dl) | 3.67 ± 0.04  0.40 (3.50 - 3.90) | 2.40-3.80 | 2.90-4.20 | 2.70-4.30 |
| Globulin (g/dl) | 3.64 ± 0.14  0.60 (3.43 - 4.03) | 3.10-5.00 | 2.10-4.30 | 2.20-4.70 |
| Total T4 (μg/dl) | 2.31 ± 0.11  0.93 (1.69 - 2.62) | 1.00-4.00 | 0.90-2.50 | 0.80-3.50 |
| BUN (mg/dl) | 35.58 ± 2.34  31.00 (22.00 - 53.00) | 15.00-32.00 | 15.40-35.00 | 16.00-44.80 |
| Creatinine (mg/dl) | 1.58 ± 0.05  0.50 (1.30 - 1.80) | 1.00-2.00 | 1.30-3.30 | 1.30-3.60 |
| ALT (U/l) | 56.45 ± 3.52  22.00 (40.75 - 62.25) | 33.00-152.00 | 36.00-132.00 | 33.00-189.00 |
| AST (U/l) | 31.73 ± 1.19  11 (26.00 - 37.25) | 1.00-37.00 | 16.00-54.00 | 15.00-72.00 |
| ALP (U/l) | 40.79 ± 2.11  17.00 (30.00 - 47.25) | 27.00-87.00 | 12.00-54.00 | 14.00-72.00 |
| Cytokine_IL1β | 1664.91 ± 216.02  3784.30 (107.42 - 3891.73) | ND |  |  |
| Cytokine_IL10 | 4.40 ± 1.31  3.25 (0.31 - 3.55) | ND |  |  |

IQR = interquartile range; WBC = total white blood cell count; ALT = alanine aminotransferase; AST = aspartate aminotransferase; ALP = Alkaline phosphatase.

Reference

1. Bellows J, Center S, Daristotle L, et al. Evaluating aging in cats: How to determine what is healthy and what is disease. *Journal of Feline Medicine and Surgery* 2016; 18: 551–570.
