# Supplementary material for: Variations in body condition score, inflammatory and metabolic biomarkers predict cognitive changes in clinically healthy senior cats
Source: Front Aging Neurosci. 2025 Nov 5;17:1703764. doi: 10.3389/fnagi.2025.1703764 (PMC12627069; doi:10.3389/fnagi.2025.1703764)
Supplement: Supplementary file 3 [file Table_3.doc]

**Supplementary Document 1:** Pre-screening questionnaire completed by caregivers interested in study participation

[*If the owner checked one or more of the conditions listed above, the recruitment process ended here. Otherwise, the owner was asked to answer the following questions.*]

***General health questions***

Please describe only what you have seen **in the last 6 months**. If you cannot answer a question for some reason, please choose NA.

0 = Never

1 = Once a month

2 = Once a week

3 = Almost every day

NA = not applicable

|  | 0 | 1 | 2 | 3 | NA |
| --- | --- | --- | --- | --- | --- |
| 1. Needs assistance to stand up |  |  |  |  |  |
| 1. Requires assistance to climb the stairs, jump on furniture, chairs, etc |  |  |  |  |  |
| 1. Limps when moving |  |  |  |  |  |
| 1. Tires quickly during exercise or play compared with previously |  |  |  |  |  |
| 1. Struggles to jump on furniture, on the bed, chairs, etc. |  |  |  |  |  |
| 1. Avoids being touched by yourself more than it used to |  |  |  |  |  |
| 1. Licks one part of the body repetitively more than it used to |  |  |  |  |  |
| 1. Has a decreased appetite |  |  |  |  |  |
| 1. Needs assistance eating, for example, hand feeding |  |  |  |  |  |
| 1. Has lost weight (with no change in diet or exercise) |  |  |  |  |  |
| 1. Has gained weight (with no change in diet or exercise) |  |  |  |  |  |
| 1. Suffers from bouts of vomiting |  |  |  |  |  |
| 1. Suffers from bouts of diarrhoea |  |  |  |  |  |
| 1. Suffers from constipation |  |  |  |  |  |
| 1. Suffers from faecal incontinence (passing faeces without being aware of it), note this does not refer to problems with house training |  |  |  |  |  |
| 1. Has an unpleasant mouth odour |  |  |  |  |  |
| 1. Drops food from their mouth when eating |  |  |  |  |  |
| 1. Flinches when eating |  |  |  |  |  |
| 1. Refuses food (and did not use to) |  |  |  |  |  |
| 1. Scratches or nibbles itself repeatedly |  |  |  |  |  |
| 1. Has urinary incontinence (passing urine without being aware of it) - note this does not refer to problems with house training or when cats urinate when excited/submissive |  |  |  |  |  |
| 1. Urinates more than it used to |  |  |  |  |  |
| 1. Drinks more than it used to |  |  |  |  |  |
| 1. Struggles passing urine / has suffered from urinary blockage |  |  |  |  |  |
| 1. Urinates outside the litterbox (and did not use to) |  |  |  |  |  |
| 1. Experienced seizures |  |  |  |  |  |
| 1. Has bouts of circling repeatedly |  |  |  |  |  |
| 1. Presses its head against surfaces |  |  |  |  |  |
| 1. Has bouts of coughing or struggling to breathe |  |  |  |  |  |
| 1. Has episodes of fainting (losing consciousness) |  |  |  |  |  |

Please choose the following statement that better describes your cat.

Hearing:

0 = He/she can hear perfectly

1 = He/she can hear most sounds

2 = He/she can hear only loud sounds

3 = He/she is completely deaf

Vision:

0 = He/she can see perfectly

1 = He/she can see but vision is somewhat limited (occasionally bumps into things, has problems identifying objects or measuring the space for a jump)

2 = His/her vision is extremely limited (they can only see shadows and exaggerated movements)

3 – He/she is completely blind

Please indicate to what degree your cat experiences the following conditions using the scoring system:

0 = No problem

1 = mildly affected

2 = moderately affected

3 = extremely affected

NA = Not applicable

|  | 0 | 1 | 2 | 3 | NA |
| --- | --- | --- | --- | --- | --- |
| 1. Has your cat shown reduced activity over the last six months (for example, playfulness, jumping on furniture)? |  |  |  |  |  |
| 1. Has your cat’s coat become greyer over the last six months? |  |  |  |  |  |
| 1. Has your cat suffered from any ear problems over the last six months (i.e., infections)? |  |  |  |  |  |
| 1. Has your cat suffered from areas of hair loss over the last six months? |  |  |  |  |  |
| 1. Does your cat have lumps on its body or in its mouth? |  |  |  |  |  |
| 1. Does your cat hold its head tilted to one side all the time? |  |  |  |  |  |
| 1. Is your cat’s fur matted / untidy/unclean (and it did not use to be)? |  |  |  |  |  |

[*Cats with a score of 0 in all categories were directly moved to the next phase of the study, the physical examination. In case of a higher score in 1 or more categories, the veterinarian contacted the owner to discuss the signs in more detail and determine if the cat was apparently healthy or not, and if it could be admitted to the physical examination phase.*]
