# Supplementary material for: Variations in body condition score, inflammatory and metabolic biomarkers predict cognitive changes in clinically healthy senior cats
Source: Front Aging Neurosci. 2025 Nov 5;17:1703764. doi: 10.3389/fnagi.2025.1703764 (PMC12627069; doi:10.3389/fnagi.2025.1703764)
Supplement: Supplementary file 4 [file Table_4.doc]

**Supplementary Document 2:** Standardized clinical examination parameters

Adapted from Bellows et al^1,2^, was performed on all cats that met pre-screening criteria to confirm the absence of clinically significant physical or behavioral signs of disease.

| **System** | **Clinically Healthy** | | **Clinically Ill** | |
| --- | --- | --- | --- | --- |
| Skin and Coat | Normal to slightly reduced self-grooming; slightly thinning and less elastic; rare small growth that does not increase in size, ulcerate, or inhibit normal activity; mild erythema or crusting around ears, nose, lips. |  | Major changes in grooming, sudden changes in coat density or shedding; unusual and rapidly growing skin lesions or growth; large patches of alopecia |  |
| Weight and Body Condition | Overweight cats with a BCS up to 7 out of 9, or underweight cats with a BCS as low as 3 out of 9 in absence of clinical signs associated. |  | Cats with BCS 8-9/9 and 1-2/9 |  |
| Musculoskeletal health | Absence of overt signs of pain, able to perform daily activities, including jumping (although this might be performed less often and at lower height). Symmetrical decrease in muscle mass over the spine, scapulae, skull, ilia, triceps and thighs. |  | Signs of pain, difficulty in performing daily activities (elimination, jumping, moving in the environment), stiff and non-fluid movement. Excessive sensitivity to the touch (withdrawn, muscle fasciculation, skin twitching). Asymmetrical loss of muscle mass. |  |
| Special senses | Slight to moderate change in vision, hearing, and smell. The effect on daily activities is moderate and manageable. Lenticular sclerosis may be present. |  | Challenges with navigating the environment, decreased response to sound. Ophthalmic and otic exam reveals abnormalities such as: Cataract, corneal ulcers, retinal degeneration, blindness. Signs of glaucoma or uveitis. Unresponsive to aversive smell (as reported by owner). |  |
| Oral and gingival health | Mild gingivitis and mild calculus with no clinical signs of pain or changes in eating/chewing, minimal mouth odor. No bleeding, pain elicited. No overt gingival recession. |  | Pain or refusal to eat, changes in chewing habits, moderate and/or severe gingivitis and calculus, severe odor. Nasal discharge. Oral masses or abnormal swelling. Bleeding or pain when exploring gingival margins. |  |
| Gastrointestinal health | No changes in defecation and stools. Healthy appetite and completion of normal rations sufficient for the cat. No GI discomfort at palpation. Neg fecal float. |  | Altered defecation or stools. Signs of GI discomfort, excessive vomiting, or diarrhoea, decreased appetite. Pain at palpation of the abdomen, abnormal liver, or bowels size/shape. Parasites on fecal float. |  |
| Cardiac health | Normal activity level and appetite. Normal HR, normal mucous membrane’s color, CRT <2 second. Normal femoral pulse. Heart murmur is associated with cardiac disease only in a small percentage of subjects, therefore cats with heart murmurs < grade III/VI will be included if there is no sign of heart disease. |  | Signs of lethargy or anorexia. Muffled heart sounds, arrhythmia, or gallop rhythm. Weak or uneven femoral pulse quality. Increased peripheral pulse quality. Blue, greyish, pale mucous membranes, CRT > 2 seconds. Cats with signs or diagnosis of heart disease cannot participate. |  |
| Respiratory health | Normal activity level. No coughing or wheezing. Normal breath sounds and rate. |  | Cough, dyspnea, wheezing, lethargy, inappetence. Increased or decreased breath sounds and/or rate. Abnormal respiratory effort. |  |
| Renal / Urinary health | Good appetite, no changes in water intake / urination. Normal to slight decrease in skin elasticity and muscle mass. Creatinine value lower than 1.6 mg/d. If higher look at IRIS/discuss with group |  | Reduced appetite, PU/PD, reduced urination, dysuria, hematuria, pollakiuria, pale mucous membranes. Abnormal kidney size or shape at palpation and/or bladder mass. |  |
| Endocrine health | Normal to non-significant changes in activity level, appetite, coat, urination. Slight decrease in skin elasticity. Muscle loss not associated with other clinical signs.  T4 values between 2.3 and 4.0 ug/dL in absence of clinical signs of hyperthyroidism. |  | Hyperactive or lethargic, increased appetite or sudden weight loss. PU/PD, hard/dry stools. Palpable enlarged/asymmetrical thyroid. T4 >  4.0 ug/dL. |  |
| Neurological health | Regular response in cranial and postural reflexes, normal mentation. |  | Head tilt, ataxia, tremors, altered reflexes, abnormal mentation. |  |

**Lab parameters**

*Snap test IDEXX Triple Feline Snap Test*

FIV test:    + ____      - ____ f

FeLV test: + ____  - ____

Heartworm: + ____ - ____

Faecal flotation:

*Complete blood count (see^1,2^ for reference intervals)*

Hematocrit

RCB

WBC

Differential count

Cytology (blood smear)

Platelets

*Serum chemistry (see^1,2^ for reference intervals)*

Urea

Creatinine

Total Ca (or tCa)

P

K^+^

Na^+^

Cl^-^

Glucose

ALT

ALP

AST

Total proteins

Albumins

Globulins

Alb/Glob

*Other*

TT4

*Biomarkers*

IL-1

IL-10

**References**

1. Bellows J, Center S, Daristotle L, et al. Aging in cats: Common physical and functional changes. *Journal of Feline Medicine and Surgery* 2016; 18: 533–550.

2. Bellows J, Center S, Daristotle L, et al. Evaluating aging in cats: How to determine what is healthy and what is disease. *Journal of Feline Medicine and Surgery* 2016; 18: 551–570.
